# The Oral Microbiota May Have Influence on Oral Cancer

**DOI:** 10.3389/fcimb.2019.00476

**Published:** 2020-01-15

**Authors:** Ling Zhang, Yuan Liu, Hua Jun Zheng, Chen Ping Zhang

**Affiliations:** ^1^Department of Oral and Maxillofacial-Head and Neck Oncology, Shanghai Ninth People's Hospital, College of Stomatology, Shanghai Jiao Tong University School of Medicine, Shanghai, China; ^2^National Clinical Research Center for Oral Diseases, Shanghai, China; ^3^Shanghai Key Laboratory of Stomatology & Shanghai Research Institute of Stomatology, Shanghai, China; ^4^NHC Key Lab of Reproduction Regulation (Shanghai Institute of Planned Parenthood Research), Fudan University, Shanghai, China

**Keywords:** oral microbiota, oral squamous cell carcinoma, *Fusobacterium nucleatum*, *Prevotella intermedia*, *Peptostreptococcus stomatis*, 16S rDNA sequencing

## Abstract

The oral microbiota plays an important role in the human microbiome and human health, and imbalances between microbes and their hosts can lead to oral and systemic diseases and chronic inflammation, which is usually caused by bacteria and contributes to cancer. There may be a relationship between oral bacteria and oral squamous cell carcinoma (OSCC); however, this relationship has not been thoroughly characterized. Therefore, in this study, we compared the microbiota compositions between tumor sites and opposite normal tissues in buccal mucosal of 50 patients with OSCC using the 16S rDNA sequencing. Richness and diversity of bacteria were significantly higher in tumor sites than in the control tissues. Cancer tissues were enriched in six families (*Prevotellaceae, Fusobacteriaceae, Flavobacteriaceae, Lachnospiraceae, Peptostreptococcaceae*, and *Campylobacteraceae*) and 13 genera, including *Fusobacterium, Alloprevotella* and *Porphyromonas*. At the species level, the abundances of *Fusobacterium nucleatum, Prevotella intermedia, Aggregatibacter segnis, Capnocytophaga leadbetteri, Peptostreptococcus stomatis*, and another five species were significantly increased, suggesting a potential association between these bacteria and OSCC. Furthermore, the functional prediction revealed that genes involved in bacterial chemotaxis, flagellar assembly and lipopolysaccharide (LPS) biosynthesis which are associated with various pathological processes, were significantly increased in the OSCC group. Overall, oral bacterial profiles showed significant difference between cancer sites and normal tissue of OSCC patients, which might be onsidered diagnostic markers and treatment targets. Our study has been registered in the Chinese clinical trial registry (ChiCTR1900025253, http://www.chictr.org.cn/index.aspx).

## Introduction

Head and neck cancers accounts for five percent of all tumors, and half of them occur specifically in the oral cavity (Kademani, 2007). Oral squamous cell carcinoma (OSCC) is a subset of head and neck squamous cell carcinoma, constituting over 90% of all oral cancers (Tandon et al., 2017). Despite advances in surgical techniques, adjuvant radiotherapy, and chemotherapy, the incidence of OSCC appears to be increasing worldwide, and the 5-year overall survival rate remains low, at approximately 50–60%. Smoking, drinking, and chewing betel are the main risk factors for oral cancer (Lin et al., 2011). Other possible risk factors may include viral infection, fungal infection, and chronic periodontitis, whereas some cases cannot be clearly explained by any known risk factors (Sanjaya et al., 2011; Hubbers and Akgul, 2015; Rischin et al., 2015; Shaikh et al., 2015).

In the 1990s, researchers demonstrated the pathogenic role of *Helicobacter pylori* in gastric cancer, linking carcinogenicity with bacteria for the first time (Marwick, 1990). Subsequently, many studies evaluated the relationships between bacteria and cancer in other organs. For example, an increased risk of gallbladder carcinoma is associated with *Salmonella typhi* infection (Scanu et al., 2015). Oral carcinogenesis is also associated with bacteria (Khajuria and Metgud, 2015). Previous studies based on bacteria culture and biochemical identification demonstrated that Gram-negative anaerobes (*Fusobacterium* spp., *Prevotella* spp., etc.) were present more frequently on the tumor surface of OSCC (Nagy et al., 1998; Bolz et al., 2014), but only semi quantitative or qualitative estimation of oral microflora were obtained. Then PCR technology and DNA-DNA hybridization were used to describe oral microflora, but each experiment could only find very limited bacterial changes (Tateda et al., 2000; Morita et al., 2003; Mager et al., 2005). With the emergence of next-generation sequencing (NGS), 16S rDNA sequencing promoted the study of associations between microbial flora and OSCC. Pushalkar et al. studied the saliva microbiome of OSCC patients and proposed their potential application as a diagnostic tool to predict oral cancer (Pushalkar et al., 2011). Zhao et al. observed that a group of periodontitis-correlated taxa was significantly enriched in OSCC samples (Zhao et al., 2017). Other studies have reported that *Fusobacterium nucleatum, Pseudomonas aeruginosa* (Al-Hebshi et al., 2017), and *Fusobacterium periodonticum* (Yang et al., 2018) are associated with OSCC development.

The control samples used for OSCC microbiota study usually include tumor adjacent or contralateral tissue, and healthy subjects (Al-Hebshi et al., 2019). The key advantage of self-normal control over healthy subjects is that the person-related factors that are easy to affect oral flora, like genotype and diet, can be avoided. During the process of oral carcinogenesis, the local microenvironment is altered (Koontongkaew, 2013). So we want to determine the microbiota composition of OSCC surface and compare it with the contralateral normal tissues. We collected samples from tumor sites and contralateral normal tissues in the buccal mucosal of 50 patients with OSCC, mainly from East China. We used strict screening criteria, made clear pathological diagnosis before sampling, and conducted careful specialized examination on the lateral anatomical position to ensure no visible lesions. Our study aimed to determine the characteristics of oral microflora on OSCC tumor sites, which has implications for further mechanistic exploration, and can be used as a biomarker to predict OSCC with high diagnostic accuracy.

## Materials and Methods

### Sample Collection

Fifty patients with SCC of the oral buccal mucosa (median age: 61 years; 63% men and 37% women) were recruited from the Department of Oral and Maxillofacial-Head and Neck Oncology of the Ninth People's Hospital (Shanghai, China), from January 2018 to July 2018. The diagnosis criteria of OSCC were confirmed by clinical presentation and pathologic diagnosis and all patients were diagnosed with OSCC for the first time and they did not have any history of cancer. For this study, we collected bilateral buccal mucosal tissues of the same patient with OSCC, thus, 100 oral tissue samples (50-paired samples) were obtained from non-tumor (50) and tumor sites (50). Patients were not on antibiotics for 1 week before sampling and had no history of other oral mucosal diseases or severe systemic disorders. Patients were prevented from drinking and eating for at least 2 h before sampling. According to a well-defined clinical protocol, the surface of the tumor site and the opposite healthy side of the oral mucosa were separately scraped 10 times. We used disposable sterile nylon flocking swab (cy-98000, Hua Chen Yang Incorporate, Shenzhen, China) for sampling and stored it in the prepared oral swab preservation solution (mainly Tris, EDTA and antiseptic) to prevent DNA degradation. All samples were kept on ice and transported to the laboratory within 2 h after collection; they were stored at −80°C at the laboratory before subsequent use. The study was approved by the Medical Ethical Committee of the Shanghai Institute of Planned Parenthood Research. Written informed consent was obtained from all participants involved in this study.

### DNA Extraction, Polymerase Chain Reaction (PCR) Amplification, and 16S rRNA Gene Sequencing

Genomic DNA was extracted using a TIANamp Swab DNA kit (Tiangen Biotech, China). The V3-4 hypervariable region of the 16S rRNA genes was amplified using the primers 338F (5′-CCTACGGGNGGCWGCAG-3′) and 806R (5′-GACTACHVGGGTATCTAATCC-3′) with a TransStart Fastpfu DNA Polymerase (TransGen, Beijing, China). Cycling conditions were as follows: 5 min at 95°C; 20 cycles of 45 s at 95°C, 30 s at 55°C, and 30 s at 72°C; and a final extension step for 10 min at 72°C. Each sample was PCR amplified in triplicate. All amplicons were purified with a QIAquick PCR Purification Kit (Qiagen, Valencia, CA, USA) and quantified on a Qubit instrument (Life Technologies). Samples were then pooled with equal concentrations, and 2 × 300 bp paired-end sequencing was performed for pooled amplicons on an Illumina MiSeq instrument.

### Bioinformatics and Statistical Analysis

Paired-end 16S rRNA gene sequences were assembled using Mothur (version 1.41.1) (Schloss et al., 2011). The following criteria were used for sequences assembly and filter: homopolymers <8, average Qscore >25, window size = 50, ambiguous bases (N) = 0, and sequence length >350 bp. The sequences alignment was performed using the SILVA reference databases (V132) (Quast et al., 2013); VSEARCH algorithm was used to identify the chimeric sequences; contaminant sequences were filtered based on the RDP trainset database, which was provided by Mothur; next, matric distances were generated, and the DNA sequences were clustered into operational taxonomic units (OTUs) at 97% similarity using the “cluster” command of Mothur. RDP classifier (80% threshold) (Wang et al., 2007) assigned the taxonomy to each OTU based on the Ribosomal Database Project (Cole et al., 2009). Assessments of community richness, evenness, and diversity (Shannon, Simpson, Shannoneven, Simpsonenven, ACE, and Chao indices and Good's coverage) were also performed using Mothur. Differences in features (taxonomy and OTUs) between the control and tumor tissues were determined using STAMP (Parks et al., 2014). Differences in bacterial diversity were assessed using analysis of molecular variance (AMOVA).

For microbiome function prediction, classification information of each OTU was generated using the classify.seqs and classify.otu command, based on OTU representative sequences, related abundance information of OTU, and GreenGenes database. Then the biom file was generated using “make.biom” of Mothur. The biom file was analyzed using the online program Phylogenetic Investigation of Communities by Reconstruction of Unobserved States (PICRUSt, http://huttenhower.sph.harvard.edu/galaxy/) (Langille et al., 2013), and KEGG pathway hierarchical categories level 3 was chosen for the predicted function analysis.

Representative sequences of OTUs were used as query sequence to define species through BlastN against HOMD RefSeq V15.1, Silva SSU database (V132), and the online NCBI database with more than 99% identity and the highest total score (Quast et al., 2013; Escapa et al., 2018).

## Results

### Bacterial Populations and Core Microbiome in Oral Samples

The clinical characteristics of the study subjects are listed in Table 1. Two oral microbiota samples (one from the OSCC lesion and one from the healthy/control site) from each patient were collected for analysis. After quality filtering, 3,267,929 16S rRNA genes (from 50 patients) were identified for subsequent analysis. The average sequencing depth was 32,679 (18,353~39,526) reads per sample. A minimum size of 18,353 was selected as a baseline for normalization to avoid statistical bias. In total, 2,983 OTUs (97% similarity) were observed from all samples. The sequencing depth (Good's coverage > 98%) was sufficient to undertake microbiota analysis with OSCC and control groups.

**Table 1:** Clinical characteristics of subjects.

	**Total**	**Male**	**Female**
**AGE**			
Mean	60.7	60.3	61.4
**Sex**	50	32 (64%)	18 (36%)
**SITES**			
Left	28	18 (36%)	10 (20%)
Right	22	14 (28%)	8 (16%)
CLINICAL STAGE^*^			
I	23	13 (26%)	10 (20%)
II	16	10 (20%)	6 (12%)
III	8	7 (14%)	1 (2%)
IV	3	2 (4%)	1 (2%)
**DRINKING**			
Previous	20	16 (32%)	4 (8%)
Current	17	14 (28%)	3 (6%)
Non-drinker	13	2 (4%)	11 (22%)
**SMOKING**			
Previous	17	15 (30%)	2 (4%)
Current	9	8 (16%)	1 (2%)
Non-smoker	24	9 (18%)	15 (30%)
**BETEL NUT CHEWING**			
Previous	4	3 (6%)	1 (2%)
Current	2	2 (4%)	0
Non-chewer	44	27 (54%)	17 (34%)

* *The criteria of clinical stage are based on the AJCC Cancer Staging Manual*.

Our analysis showed that 99.0% of the oral microbiota was aligned into 13 phyla. Additionally, 95.6% of the oral microbiota was clustered into 82 families, and 91.0% was aligned into 162 genera. At the phylum level, the common bacteria *Firmicutes, Proteobacteria, Bacteroidetes, Fusobacteria*, and *Actinobacteria* were dominant in both the OSCC and control groups. At the family level, 17 families were identified as the major taxa and core microbiota co-existing in the OSCC and control groups, accounting for over 91.2% of the microbiome in both groups (Table 2). Among the 17 families, *Streptococcaceae, Prevotellaceae, Neisseriaceae, Pasteurellaceae, Fusobacteriaceae* and *Veillonellaceae* were dominant (> 69.7% of the entire microbiome). Among the 162 genera, 41 were the dominant genera (with each genus comprising >0.1% of the total microbiome), including *Streptococcus, Neisseria, Haemophilus*, and *Prevotella* (Table 3). Among the 41 dominant genera, 11 ubiquitous (core) genera were consistently found in all samples and comprised more than 62.4% of the total microbiome.

**Table 2:** Dominant families and significant differences between the OSCC and Control groups computed by STAMP.

**Phylum**	**Family**	**OSCC**	**Control**	**Enriched in**
Firmicutes	Streptococcaceae	12.13%	25.54%	Control
Bacteroidetes	Prevotellaceae	17.85%	11.99%	OSCC
Proteobacteria	Pasteurellaceae	12.57%	12.55%	
Proteobacteria	Neisseriaceae	9.92%	10.36%	
Fusobacteria	Fusobacteriaceae	11.03%	3.29%	OSCC
Firmicutes	Veillonellaceae	5.25%	6.87%	
Fusobacteria	Leptotrichiaceae	4.21%	3.31%	
Bacteroidetes	Porphyromonadaceae	3.47%	2.47%	
Bacteroidetes	Flavobacteriaceae	3.65%	1.76%	OSCC
Actinobacteria	Micrococcaceae	1.32%	3.73%	Control
Firmicutes	Bacillales_Incertae Sedis XI	1.91%	2.53%	
Proteobacteria	Burkholderiaceae	0.88%	2.83%	
Firmicutes	Lachnospiraceae	2.12%	1.24%	OSCC
Firmicutes	Peptostreptococcaceae	1.92%	0.41%	OSCC
Proteobacteria	Campylobacteraceae	1.66%	0.61%	OSCC
Actinobacteria	Actinomycetaceae	0.64%	1.61%	Control
Firmicutes	Carnobacteriaceae	0.67%	1.54%	Control

**Table 3:** Dominant genera and significant differences between the OSCC and Control groups computed by STAMP.

**Phylum**	**Genus**	**OSCC**	**Control**	**Feature**
Firmicutes	Streptococcus	12.10%	25.50%	Ubiquitous (core)
Proteobacteria	Haemophilus	8.65%	10.82%	
Bacteroidetes	Prevotella	11.02%	7.92%	Ubiquitous (core)
Proteobacteria	Neisseria	7.92%	8.96%	Ubiquitous (core)
Fusobacteria	Fusobacterium	10.98%	3.27%	Ubiquitous (core)
Firmicutes	Veillonella	3.05%	5.33%	Ubiquitous (core)
Fusobacteria	Leptotrichia	4.04%	3.25%	
Bacteroidetes	Alloprevotella	4.79%	2.30%	Ubiquitous (core)
Bacteroidetes	Porphyromonas	3.13%	1.95%	Ubiquitous (core)
Actinobacteria	Rothia	1.32%	3.72%	Ubiquitous (core)
Bacteroidetes	Capnocytophaga	3.43%	1.58%	Ubiquitous (core)
Firmicutes	Gemella	1.91%	2.53%	Ubiquitous (core)
Proteobacteria	Lautropia	0.88%	2.83%	
Proteobacteria	Aggregatibacter	2.59%	0.92%	
Proteobacteria	Campylobacter	1.66%	0.61%	
Firmicutes	Granulicatella	0.67%	1.53%	Ubiquitous (core)
Actinobacteria	Actinomyces	0.59%	1.43%	
Firmicutes	Selenomonas	1.31%	0.70%	
Candidatus Saccharibacteria	Saccharibacteria _genera _incertae _sedis	0.67%	1.30%	
Spirochaetes	Treponema	1.29%	0.38%	
Firmicutes	Peptostreptococcus	1.18%	0.22%	
Firmicutes	Lachnoanaerobaculum	0.61%	0.40%	
Firmicutes	Peptococcus	0.79%	0.16%	
Firmicutes	Catonella	0.81%	0.12%	
Actinobacteria	Corynebacterium	0.20%	0.66%	
Firmicutes	Dialister	0.49%	0.17%	
Bacteroidetes	Tannerella	0.20%	0.44%	
Firmicutes	Parvimonas	0.46%	0.12%	
Firmicutes	Filifactor	0.36%	0.14%	
Firmicutes	Solobacterium	0.31%	0.12%	
Proteobacteria	Morococcus	0.16%	0.25%	
Firmicutes	Peptostreptococcaceae _incertae _sedis	0.36%	0.04%	
SR1	SR1_genera _incertae _sedis	0.21%	0.18%	
Firmicutes	Oribacterium	0.12%	0.23%	
Firmicutes	Stomatobaculum	0.10%	0.21%	
Proteobacteria	Pseudomonas	0.25%	0.04%	
Actinobacteria	Atopobium	0.18%	0.10%	
Firmicutes	Megasphaera	0.08%	0.20%	
Firmicutes	Abiotrophia	0.15%	0.08%	
Firmicutes	Lactobacillus	0.07%	0.13%	
Proteobacteria	Cardiobacterium	0.06%	0.14%	

### Changes in Bacterial Composition Between the OSCC and Control Groups

**Figure 1:**
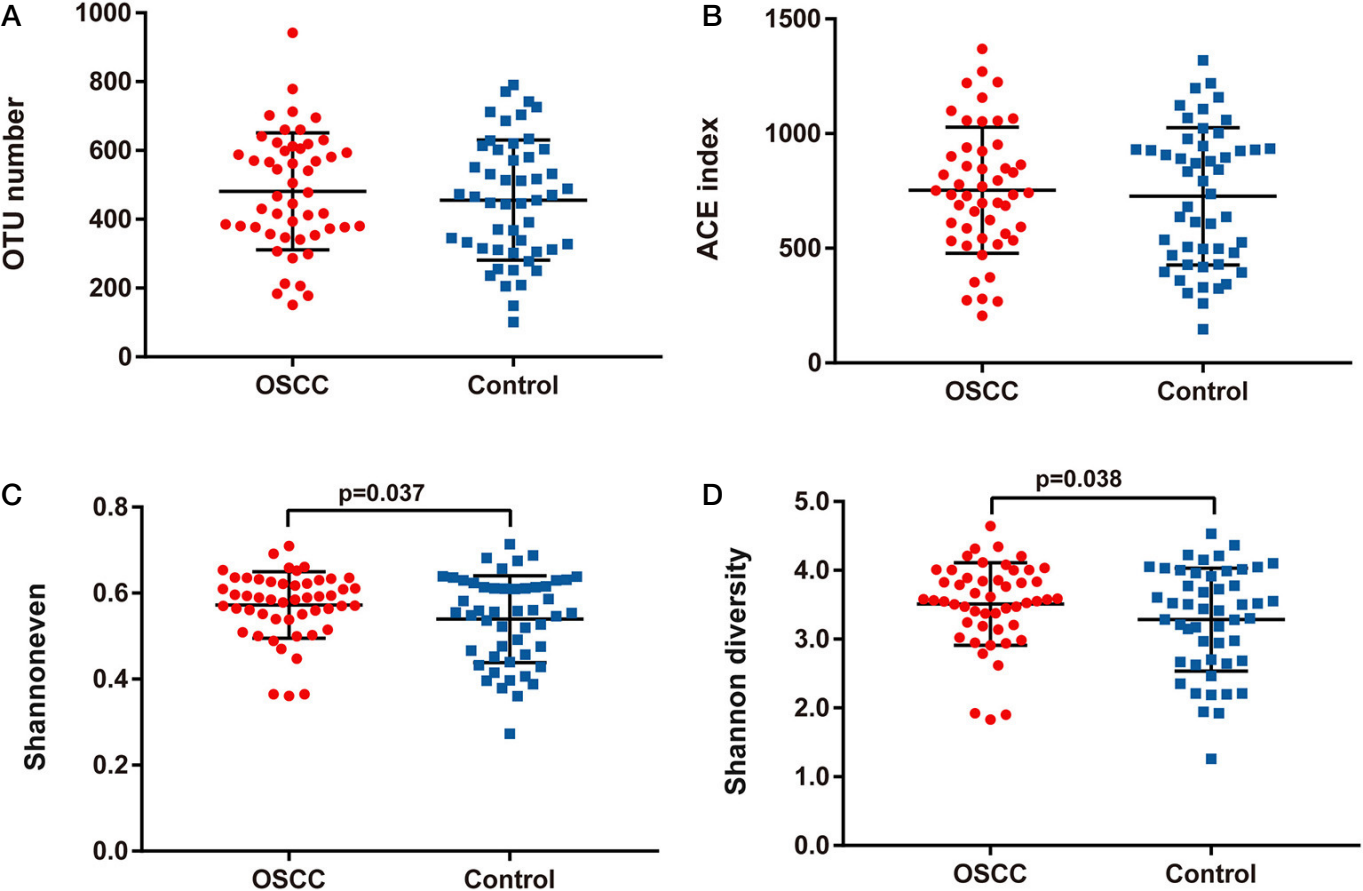
Comparison of bacterial richness, evenness and diversity between OSCC and Control groups. **(A)** OTU number, **(B)** ACE index, **(C)** Shannon even index and **(D)** Shannon diversity index.

**Figure 2:**
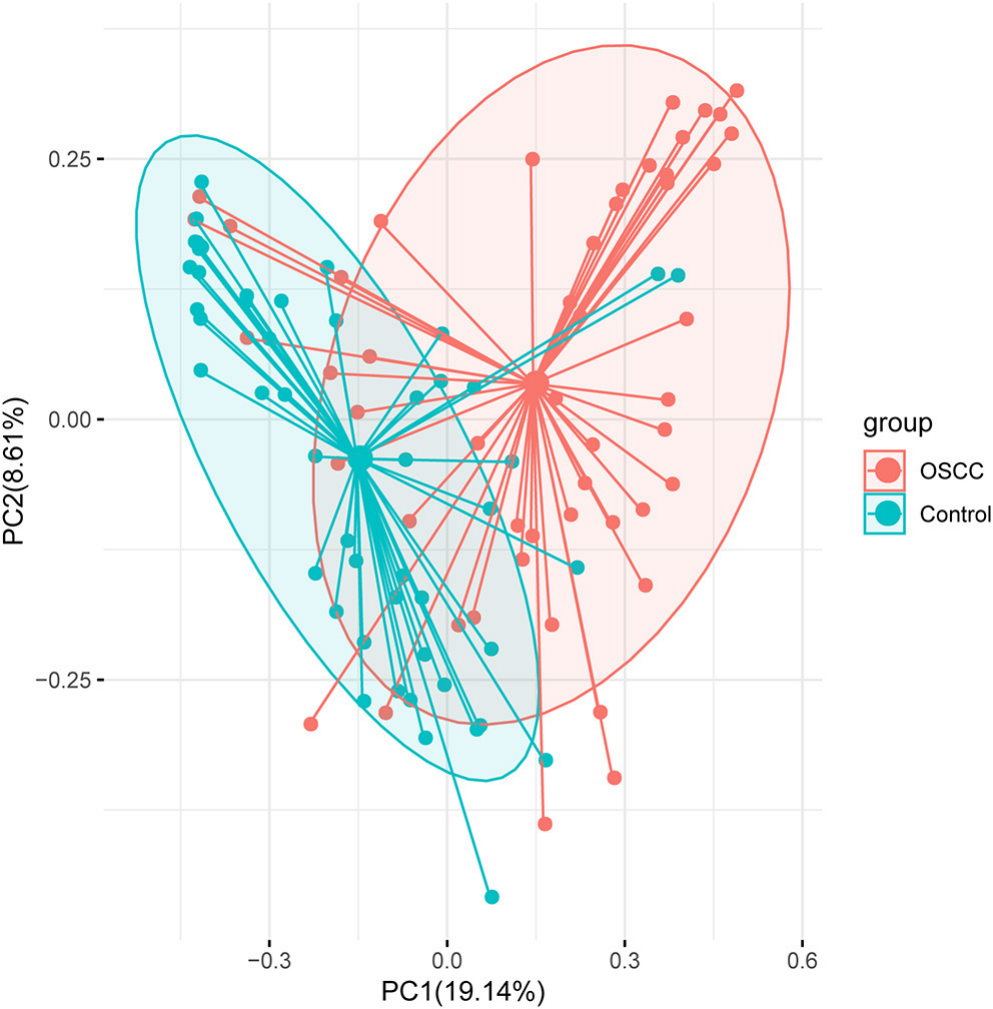
Principal component analysis (PCA) analysis with Bray-Curtis dissimilarity based on genera between the microbiota of the two groups. Points represent samples. Samples that are more similar to one another are ordinated closer together. The groups show significant differences in similarity tested by ANOSIM (*P*_ANOSIM_ < 0.001).

We compared the oral microbiota profiles of the OSCC and control groups. AMOVA showed significant differences in microbiota between the two groups (*P*_AMOVA_ < 0.001). Species evenness and diversity were significantly higher in the OSCC group than in the control group (Figure 1). Principal component analysis (PCA) was conducted to visualize the different diversities of microbiota in the two groups (Figure 2).

Among the 17 major families, 10 families showed significant differences (*P* < 0.05) between the OSCC and control groups. The families *Streptococcaceae, Micrococcaceae Actinomycetaceae* and *Carnobacteriaceae* were decreased in the OSCC group, whereas another six families were increased in the OSCC group (Table 2). Both *Neisseriaceae* and *Pasteurellaceae* were present at over 10% in the OSCC and control groups, without significant differences between groups. Thus, these two families were stable and common microbiota in the oral cavity.

**Figure 3:**
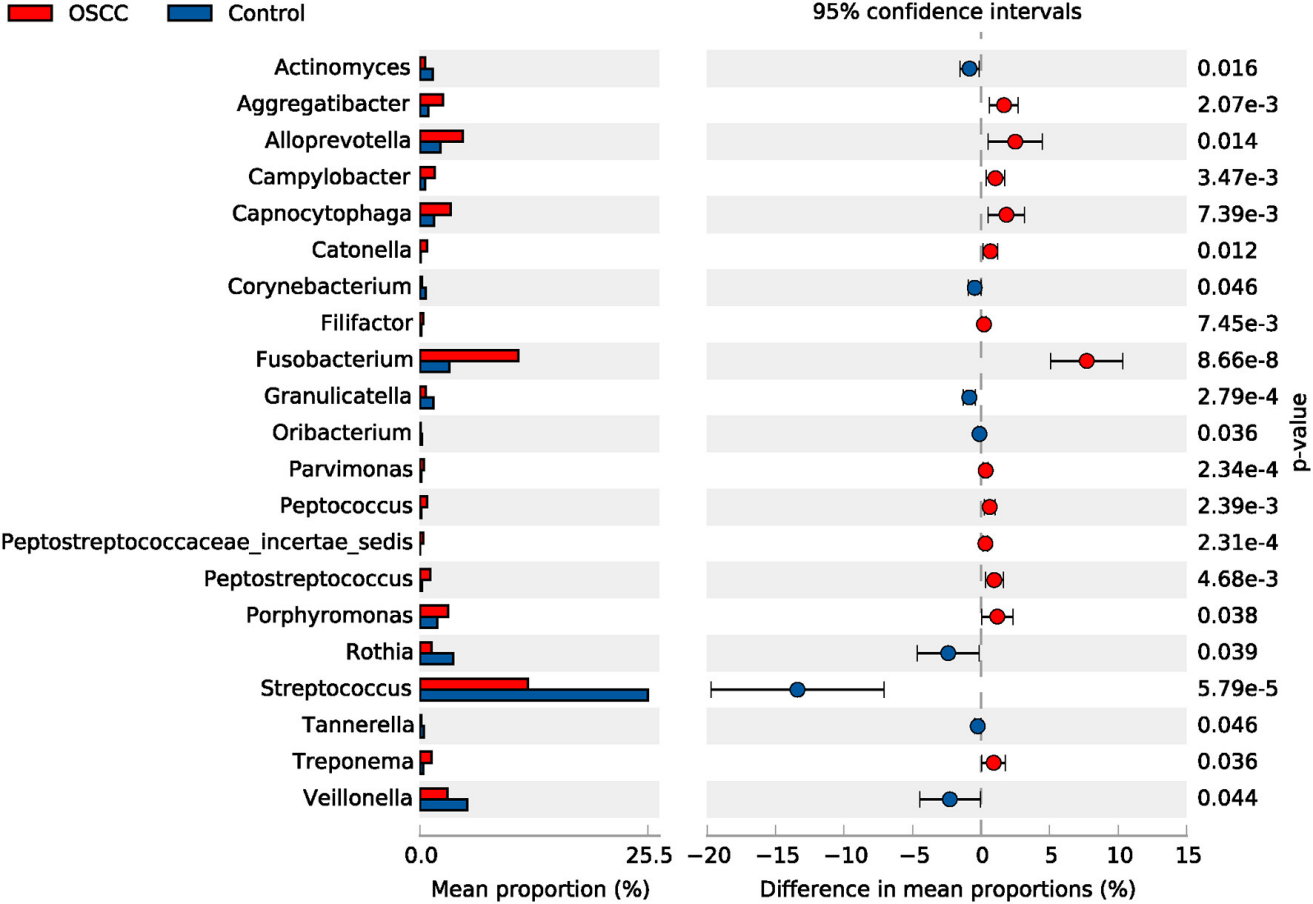
Comparative taxonomic profile of the OSCC and Control groups at genus level. The genera with significant richness difference (*P* < 0.05, computed by STAMP) between the two groups are shown.

Among the 41 dominant genera, 21 genera showed significant differences (*P* < 0.05) between the OSCC and control groups. Eight genera, including *Streptococcus, Veillonella* and *Rothia*, showed significant decreases in the OSCC group (*P* < 0.05), whereas 13, including *Fusobacterium, Alloprevotella* and *Porphyromonas*, showed significant increases (Table 3, Figure 3). The genus *Neisseria, Prevotella* and *Gemella* were stable and common microbes (occupying about 20% in both the OSCC and control groups). Taken together, the results of these analyses indicated that the composition of the core bacteria present in the oral cavity was significantly altered in OSCC.

**Figure 4:**
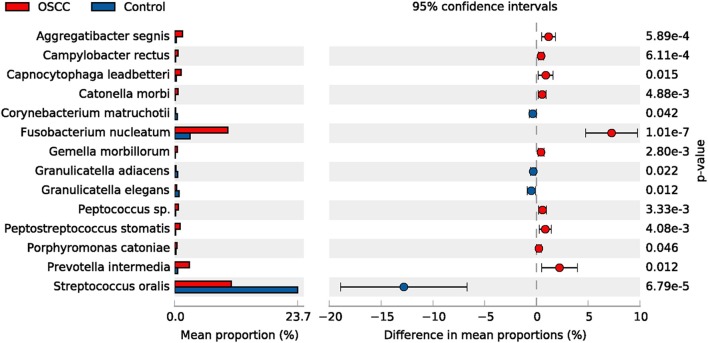
Comparative taxonomic profile of the OSCC and Control groups at species level. The species with significant richness difference (*P* < 0.05, computed by STAMP) between the two groups are shown.

The top 50 species (OTUs) were chosen to identify differentially enriched species within groups using STAMP. In total, 14 species showed differences between two groups, such as *Streptococcus oralis*. Four species were decreased, whereas 10 species were increased in the OSCC group (Figure 4).

### Predicted Functional Changes in the Microbiomes of the OSCC and Control Groups

**Figure 5:**
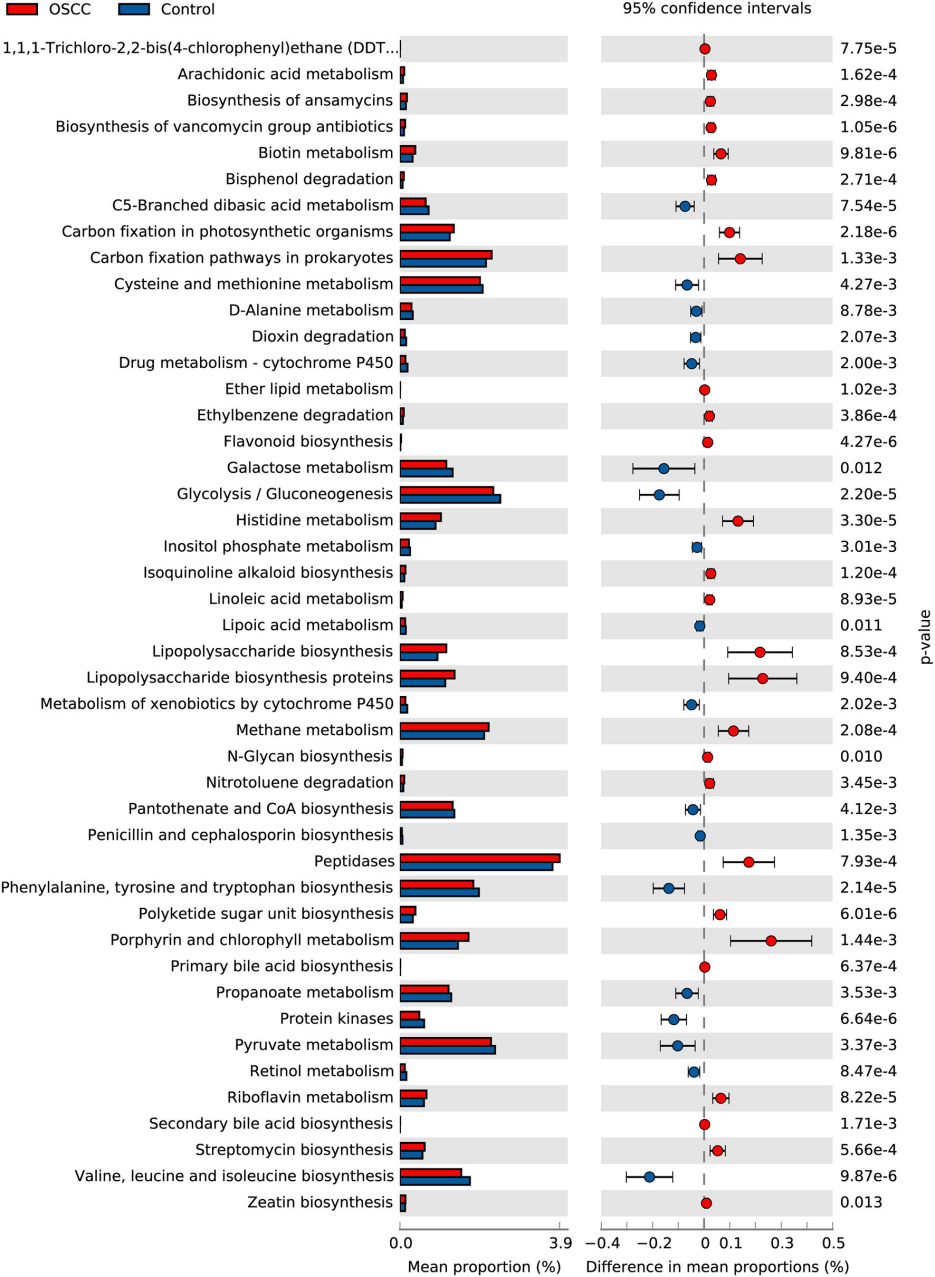
Comparative functional profile of oral microbiota between OSCC and Control groups. Microbial functions were predicted using PICRUSt at the third level of the KEGG pathway, and statistically analyzed by STAMP. KEGG pathways with significant abundance difference (*P* < 0.05) are shown.

We used PICRUSt to predict and compare potential changes in microbial functions between the two groups. In the metabolism category, 45 Metabolism pathways and 14 pathways related to Genetic Information Processing were identified as having significant differences (*P* < 0.05) between the OSCC and control groups (Figure 5). Analysis revealed the relative abundance of genes associated with proinflammatory bacterial component, such as lipopolysaccharide biosynthesis; and genes involved in metabolism of cofactors and vitamins, such as Porphyrin and chlorophyll metabolism, were significantly increased in cancer sites. Genes participating in carbohydrate metabolism and PTS transport were significantly decreased. Other pathways, in particular the genes related to cell motility, such as bacterial chemotaxis and flagellar assembly, were remarkably enriched in the OSCC group.

## Discussion

As a part of the digestive tract, oral cavity includes diverse microorganisms (Segata et al., 2012), and oral microbiota is a complex microbial community (Lamont et al., 2018). The oral microbiota plays an important role in human health, and dysbiosis of oral microbiota can lead to a variety of systemic diseases (Olsen and Yamazaki, 2019). Since changes in gut microbial composition may contribute to cancer initiation and progression (Vivarelli et al., 2019), oral microbial dysbiosis may also be involved in the occurrence and development of oral cancer.

**Figure 6:**
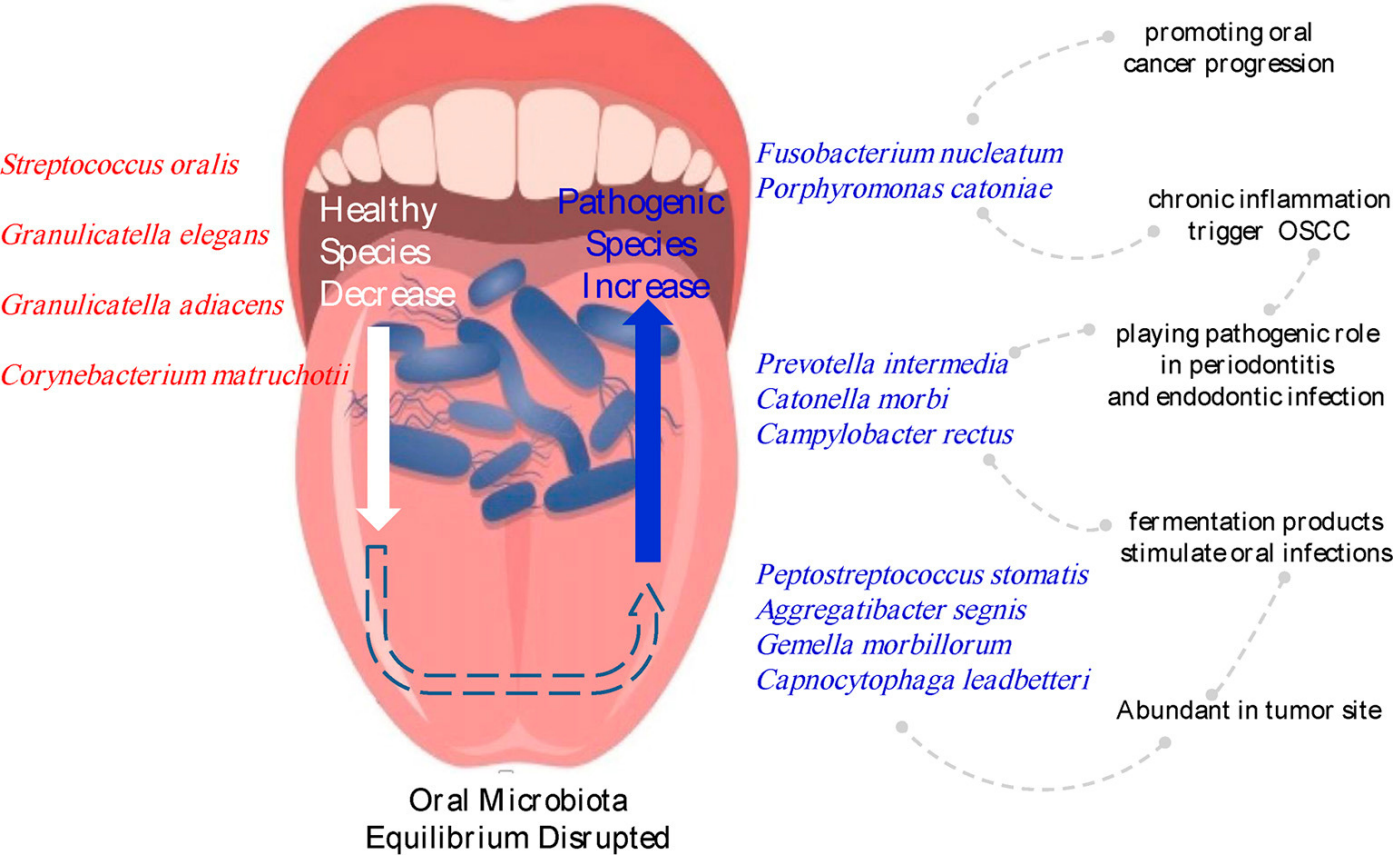
Changes in the microbiota composition associated with OSCC. Species names labeled with red indicate bacteria enriched in normal sites, and species names labeled with blue indicate bacteria increased in tumor sites.

In this study, we aimed to determine the relationships between oral buccal mucosal microbial profile and OSCC. Through the analysis, we found significant changes in microbiota between tumor sites and contralateral normal tissues in the buccal mucosa. In terms of the composition of the oral microbiota, *Firmicutes, Proteobacteria, Bacteroidetes, Fusobacteria*, and *Actinobacteria* were the five dominant phyla in the mouth. Six families (*Prevotellaceae, Fusobacteriaceae, Flavobacteriaceae, Lachnospiraceae, Peptostreptococcaceae*, and *Campylobacteraceae*) and 13 genera, including *Fusobacterium, Alloprevotella* and *Porphyromonas*, were enriched in cancer tissues, whereas *Streptococcus, Veillonella*, and *Rothia*, were significantly decreased in cancer tissues. Ten species showed significantly increased abundances in cancer lesions. These species included *Fusobacterium nucleatum, Prevotella intermedia, Aggregatibacter segnis, Peptostreptococcus stomatis*, and *Catonella morbi*, which reside in the oral mucosa as commensals but may be opportunistic pathogens with potential correlations with OSCC (Figure 6).

*Fusobacterium nucleatum*, a known pathogenic oral species showing a 5.88% increase in cancer lesions, has been reported to enhance oral cancer progression via direct interactions with oral epithelial cells (Binder Gallimidi et al., 2015). Al-hebshi reported the associations of *F. nucleatum* and *Pseudomonas aeruginosa* with OSCC (Al-Hebshi et al., 2017). However, in our analysis, *P. aeruginosa* was not enriched in cancer lesions. A metatranscriptome analysis revealed that *Fusobacteria* was the main phylum causing increased expression of virulence factors in the oral microbiome of OSCC patients, and *F. nucleatum* was the most active bacterium expressing putative virulence factors in the tumor sites (Yost et al., 2018). *F. nucleatum* infection is prevalent in human colorectal carcinoma (Castellarin et al., 2012), and various mechanisms are involved in the process. For example, *F. nucleatum* can suppress host immunity leading to the carcinogenesis of colorectal cancer. *F. nucleatum* inhibits T cells and NK cells function by directly interacts with human CEACAM1 (Gur et al., 2019; Wu et al., 2019).

*Prevotella intermedia* and *Porphyromonas gingivalis* are considered to be the pathogen of periodontitis (Mysak et al., 2014; Zhang et al., 2017; Hsiao et al., 2018). Studies have reported that repeated periodontitis were associated with increased risk of OSCC (Li et al., 2018; Shin et al., 2019). Both of them can secrete peptides (Lisi et al., 2014; Zhang et al., 2017; Eftekhari et al., 2018). It is reported that proteases can act as signaling molecules through activation of proteinase-activated receptors (PARs) (Van Spaendonk et al., 2017), which involves cell proliferation and apoptosis, autoimmunity (Lisi et al., 2014), cytokine production, microenvironment inflammation, pain and epithelial barrier function (Amadesi and Bunnett, 2004). There was an increase of peptidases in tumor sites by functional analysis. Proteases produced by bacteria can degrade host tissue like extracellular matrix (ECM), destruct host physical barriers, and modulate host immune response, finally contributing to the onset and progression of tumors (Alfano et al., 2016).

Several bacterial inflammatory processes, such as lipopolysaccharide synthesis, flagella assembly, and bacterial chemotaxis, also have roles in mediating inflammation in cancer (Al-Hebshi et al., 2017; Perera et al., 2018). Chronic inflammation of the oral cavity, usually caused by microorganisms, has been observed at various stages of OSCC (Pushalkar et al., 2011; Chen et al., 2017). Poor oral hygiene can also lead to periodontitis, and many studies have shown that poor periodontal health status, such as gingivitis and periodontitis, can be a direct or indirect risk factor for oral cancer (Tezal et al., 2009; Perera et al., 2016). Thus, disruption of the balance between microbes and human hosts can increase the risk of many diseases, including cancer, regardless of external factors (such as the use of alcohol and cigarettes) or pathogenic microbial infections.

Lipopolysaccharide (LPS), composed of lipids and polysaccharides, is a component of the cell wall of Gram-negative bacteria. Functional analysis in our study revealed a significant increase in LPS biosynthesis in OSCC sites. LPS has been reported to enhance OSCC progression and migration (Kurago et al., 2008; He et al., 2015). In innate and adaptive immunity, LPS is recognized by LPS binding protein (LBP) and Toll-like receptor 4 (TLR4) to stimulate cytokine transcription (Park and Lee, 2013) as part of the recognition of pathogen-associated molecular patterns (PAMPs) (Kumagai and Akira, 2010), thus causing the LPS-induced inflammation. In T cells, TLR4-ligand LPS stimulated the TLR directing the cells toward type 1 polarization and expressed suppressor of cytokine signaling (SOCS) 1, and thus suppressed IL-10 expression (Ghosh et al., 2015). IL-10 was considered as a switch from tumor-promoting inflammation to antitumor immunity, and deficient IL-10 signaling developed tumors spontaneously and at high rates (Oft, 2014; Talero et al., 2016). LPS could activate TLR4 signaling in tumor cells and help tumor cells escape attack from cytotoxic lymphocyte (CTL) and natural killer (NK) cells (Huang et al., 2005).

The functional prediction of oral bacterial communities also revealed enrichment of genes involved in bacterial chemotaxis and flagellar assembly. This result is consistent with the report of Al-Hebshi et al. who performed functional analysis of the microbiome associated with OSCC based on the V1-V3 region of 16s rDNA, and proposed that bacterial flagella is a potent inflammatory structure like LPS, and bacterial chemotaxis play an important role in cancer-related inflammation (Al-Hebshi et al., 2017). The decrease in the phosphotransferase system (PTS), glycolysis and galactose metabolism might reflect the community response to reduced sugar source on the tumor surface, since increased glucose uptake is essential for OSCC cells to survive (Eckert et al., 2016).

We also emphasized the relationship between bacteria, chronic inflammation, and tumors. Inflammation is a defensive response, which restores tissue injury and eliminates pathogenic agents. Transient inflammation is thought to be part of the body's immune defenses against pathogens, but persistent inflammation can lead to cancer (Mirjalili and Kheirollahi, 2014; Crusz and Balkwill, 2015). Once the balance in bacterial communities is broken, the dominance of pathogens or a significant increase in biomass will result in an inflammatory defense response in the human body. For example, *Porphyromonas*, especially *Porphyromonas gingivalis*, are obligatorily anaerobic, and their fermentation end products are associated with chronic inflammation (Gibson and Genco, 2006). However, if inflammation is unregulated or continuous, it can become chronic, which can induce malignant cell transformation in the surrounding tissue. This process involves a variety of inflammatory factors and signaling pathways, as a result, chronic inflammation works in carcinogenesis, tumor growth, epithelial mesenchymal transition (EMT), angiogenesis, and metastasis (Landskron et al., 2014). The tumor microenvironment is hypoxic; most of the bacteria that changed significantly in oral cancer were anaerobes. Pathogenic bacteria can promote the occurrence and development of malignant tumors. The tumor microenvironment can selectively promote the growth of specific bacteria.

We should not neglect taxa with low abundance but showing significant increases in OSCC because these taxa might be taken as “keystone” microbes and may have stronger virulence and thus play a greater role in the development of cancer. The genus *Peptostreptococcaceae incertae sedis*, occupying only 0.04% in normal oral buccal mucosa, increased to 0.36% in OSCC sites. *Catonella morbi*, a Gram-negative anaerobic bacillus, is involved in primary endodontic infections (Siqueira and Rocas, 2006). Increased abundance of *Catonella* spp. and *Catonella morbi* in patients with chronic obstructive pulmonary disease (COPD) and periodontitis compared with that in patients without COPD (Wu et al., 2017) suggests that this periodontitis-associated bacteria may be related to oral cancers. *Gemella morbillorum*, a facultative anaerobic Gram-positive coccus of the phylum *Firmicutes*, is highly associated with OSCC tumor sites (Pushalkar et al., 2012) and has been cultured from both deep-tissue specimens and corresponding superficial tissues of OSCC samples (Hooper et al., 2006). *Campylobacter rectus* also plays pathogenic role in human periodontitis (Rams et al., 1993), and chronic inflammation may be a possible trigger for OSCC (Crusz and Balkwill, 2015). In a previous case report, a patient with OSCC was reported to also be suffering from advanced chronic periodontitis infected with *Campylobacter rectus, Porphyromonas gingivalis, Peptostreptococcus micros*, and *Fusobacterium nucleatum* (Kruger et al., 2013).

In summary, we found that OSCC tissues exhibited a unique microbiota compared with contralateral normal tissues. From our findings, we propose that recolonization of bacteria may disrupt the equilibrium between the resident oral microbiota and the host. This may be a key link through which commensal oral bacteria promote oral cancer. Accordingly, these findings may provide insights into the development of vaccines and/or antimicrobial therapies to prevent OSCC. Alternatively, the significant association of bacteria with OSCC may have clinical utility in screening for cancer. Further studies are needed to explore these possibilities.

## Data Availability Statement

The sequence data have been submitted to the NCBI Sequence Read Archive (Accession Number: PRJNA533177).

## Ethics Statement

The studies involving human participants were reviewed and approved by the Medical Ethical Committee of Shanghai Institute of Planned Parenthood Research. The patients/participants provided their written informed consent to participate in this study.

## Author Contributions

LZ, YL, and HZ contributed to the conception, design, data acquisition, analysis, and interpretation, drafted and critically revised the manuscript. CZ contributed to the conception, design, data analysis, and interpretation, and critically revised the manuscript. All authors gave final approval and agree to be accountable for all aspects of the work.

### Conflict of Interest

The authors declare that the research was conducted in the absence of any commercial or financial relationships that could be construed as a potential conflict of interest.
